# Changes in White Matter of the Cervical Spinal Cord after a Single Season of Collegiate Football

**DOI:** 10.1089/neur.2020.0035

**Published:** 2021-02-17

**Authors:** Brian Johnson, Alexa E. Walter, James R. Wilkes, Linda Papa, Semyon M. Slobounov

**Affiliations:** ^1^ Department of Radiology, University of Texas Southwestern Medical Center, Dallas, Texas, USA.; ^2^ Department of Kinesiology, The Pennsylvania State University, University Park, Pennsylvania, USA.; ^3^ Department of Emergency Medicine, Orlando Regional Medical Center, Orlando, Florida, USA.; ^4^ Department of Neurology and Neurosurgery, McGill University, Montreal, Quebec, Canada.

**Keywords:** cervical spinal cord, diffusion tensor imaging, football, head acceleration events, subconcussion

## Abstract

The involvement of the central nervous system (CNS), specifically the white matter tracts in the cervical spinal cord, was examined with diffusion tensor imaging (DTI) following exposure to repetitive head acceleration events (HAEs) after a single season of collegiate football. Fifteen National Collegiate Athletic Association (NCAA) Division 1 football players underwent DTI of the cervical spinal cord (vertebral level C1–4) at pre-season (before any contact practices began) and post-season (within 1 week of the last regular season game) intervals. Helmet accelerometer data were also collected in parallel throughout the season. From pre-season to post-season, a significant decrease (*p* < 0.05) of axial diffusivity was seen within the right spino-olivary tract. In addition, a significant decrease (*p* < 0.05) in global white matter fractional anisotropy (FA) along with increases (*p* < 0.05) in global white matter mean diffusivity (MD) and radial diffusivity (RD) were found. These changes in FA from pre-season to post-season were significantly moderated by previous concussion history (*p* < 0.05) and number of HAEs over 80 g (*p* < 0.05). Despite the absence of sports-related concussion (SRC), we present measurable changes in the white matter integrity of the cervical spinal cord suggesting injury from repetitive HAEs, or SRC, may include the entirety of the CNS, not just the brain.

## Introduction

Sports-related concussion (SRC) is a traumatic brain injury originating from the transmission of biomechanical forces to the head following exposure to impacts anywhere on the body.^1^ Its mechanism of injury shares many similarities with whiplash-associated disorders (WADs)^2^ highlighting the vulnerability of the cervical spine to injury.^3,4^ Consequently, there is a large overlap in their symptomology; however, for most SRC, clinical symptoms resolve in 10–14 days^1^ in stark comparison to WAD where upwards of 50% of individuals report symptoms 1 year post-injury.^5^ Further, it is clinically accepted that there is comorbid cervical injury from SRC^3^ and any damage to the cervical spinal cord may have direct influence on vestibular, visual, and/or somatosensory function.^2^ Despite the anatomical proximity and function of the spinal cord in the central nervous system (CNS), there is a paucity of research regarding the involvement of the cervical spinal cord in SRC,^6^ whereas the interaction of the head and soft-tissue neck in SRC has been greatly debated and studied.^7^

Head acceleration events (HAEs) involve the transfer of mechanical energy at enough force to injure axonal or neuronal integrity,^8^ while not eliciting any clinical symptoms,^9^ and are capable of causing cervical injuries.^3^ In addition, contact sports have an inherent risk of exposure to repetitive impacts, and it has been shown that athletes involved in contact sports can be exposed to upwards of 1000 HAEs during the course of a season.^8,10^

Diffusion tensor imaging (DTI) has become a recommended technique to objectively quantify white matter microstructure by assessing changes in diffusivity and fractional anisotropy (FA) from SRC^11,12^ and exposure to HAEs.^13–15^ However, all of the HAEs literature to date has concentrated exclusively on the brain.^16^ The spinal cord is a vital component in relaying sensory and motor information,^2^ yet research assessing its integrity following SRC and HAEs using advanced neuroimaging is lacking. Therefore, the objective of this study was to examine the influence of repetitive HAEs on white matter tracts of the cervical spinal cord (vertebral level C1–4) over the course of one collegiate football season using DTI. Helmet accelerometer data were also recorded to evaluate the relationship between HAEs and their effects on spinal cord integrity.

To our knowledge, this is the first study to examine the role of the cervical spinal cord in relation to repetitive HAEs. We hypothesized that there would be subtle changes in DTI metrics in the form of decreased FA and axial diffusivity (AD) along with increased mean diffusivity (MD) and radial diffusivity (RD) when comparing pre-season with post-season scans indicating injured white matter tracts.^17,18^ Moreover, the extent of these changes would be influenced by the volume of HAEs sustained over the course of a season. Given the diffuse nature of SRC injury in the brain, we also speculated that both afferent and efferent white matter tracts would be affected from HAEs exposure and would be modulated by demographic factors known to affect outcome (position played, number of years played, and previous concussion history).

## Methods

### Design, setting, and participants

This was a prospective, observational study conducted from July to December 2015. Fifteen Pennsylvania State University Football Bowl Subdivision players were studied over the course of a single season and completed both pre-season and post-season scans. Pre-season scans were acquired before any contact practice began for the season and post-season scans were finished within 1 week of the last regular season game. No athletes under study were diagnosed with a concussion during the season. All participants provided written informed consent, as approved by the Pennsylvania State University Institutional Review Board and in accordance with the Declaration of Helsinki.

### Head acceleration event assessment

Impacts to the head were monitored using Head Health Network's BodiTrak system. The sensors are individually placed in each athlete's helmet using 3M VHB adhesive and are comprised of elastic fiber with pressure monitors and impact sensors. Output includes linear acceleration (in units of G) and data were collected over the course of the season during practices.

### MRI acquisition

**FIG. 1.**
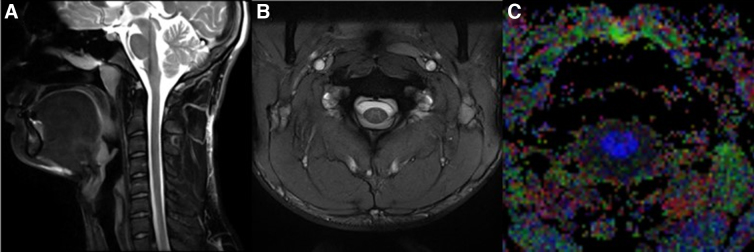
Acquired scans. **(A)** 2D sagittal T2-weighted fat saturated turbo spin echo, **(B)** 3D axial T2*-weighted multi-echo gradient echo, and **(C)** 2D axial diffusion tensor imaging. 2D, two-dimensional; 3D, three-dimensional.

Magnetic resonance imaging (MRI) scans were performed on a Siemens 3T Prisma MR scanner (Siemens, Erlangen, Germany) with a 20-channel head/neck coil. Identical imaging protocols were obtained at both pre-season and post-season that included the following sequences (Fig. 1): two-dimensional (2D) sagittal T2-weighted fat-saturated turbo spin echo, three-dimensional (3D) axial T2*-weighted multi-echo gradient echo, and 2D axial diffusion-weighted imaging. Sagittal T2-weigthed images were acquired (echo time [TE] = 92 msec, repetition time [TR] = 3520 msec, resolution = 0.7 × 0.7 × 3.0 mm, slices = 15, fat saturation = spectral attenuated inversion recovery [SPAIR], acquisition time [TA] = 1:58) to evaluate the cervical spine for any surrounding soft-tissue or ligamentous injury.

**FIG. 2.**
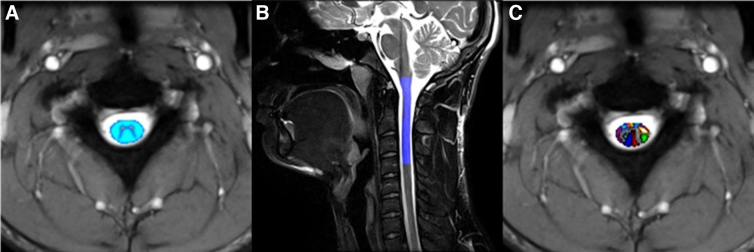
Example of post-processing. **(A)** White matter segmentation, **(B)** registration, and **(C)** warping of white matter atlas with 30 spinal tract regions of interest.

Axial multi-echo gradient echo images (TE = 17 msec, TR = 530 msec, resolution = 0.5 × 0.5 × 4.0 mm, slices = 20, magnetization transfer = yes, TA = 7:14) were acquired to provide superior gray–white matter contrast for segmentation and registration. DTI metrics were computed from diffusion-weighted images acquired with a multi-segmented readout spin-echo echo-planar imaging sequence (TE = 63 msec, TR = 3000, resolution = 1.0 × 1.0 × 4.0 mm, slices = 20, b-values = 0 and 600 sec/mm^2^, directions = 20, TA = 10:41) to assess white matter integrity. Image analysis was performed with Spinal Cord Toolbox^19^ and consisted of spinal cord segmentation, affine and non-linear registration to the MNI-Poly-AMU template,^20^ and warping of white matter atlas to template (Fig. 2).

White matter atlas consists of 30 white matter tracts (Table 1). DTI images were motion corrected,^21^ co-registered, and FA (a simplistic measure of structural integrity of white matter), MD (a simplistic measure of molecular diffusion rates or membrane density), AD (a simplistic measure of axonal degeneration), and RD (a simplistic measure of myelination) metrics were computed.^22^

**Table 1.** Spinal Cord White Matter (WM) Tracts

ROI	ROI label name	Efferent or afferent
1	WM left fasciculus gracilis	Afferent
2	WM right fasciculus gracilis	Afferent
3	WM left fasciculus cuneatus	Afferent
4	WM right fasciculus cuneatus	Afferent
5	WM left lateral corticospinal tract	Efferent
6	WM right lateral corticospinal tract	Efferent
7	WM left ventral spinocerebellar tract	Afferent
8	WM right ventral spinocerebellar tract	Afferent
9	WM left rubrospinal tract	Efferent
10	WM right rubrospinal tract	Efferent
11	WM left lateral reticulospinal tract	Efferent
12	WM right lateral reticulospinal tract	Efferent
13	WM left spinal lemniscus (spinothalamic and spinoreticular tracts)	Afferent
14	WM right spinal lemniscus (spinothalamic and spinoreticular tracts)	Afferent
15	WM left spino-olivary tract	Afferent
16	WM right spino-olivary tract	Afferent
17	WM left ventrolateral reticulospinal tract	Efferent
18	WM right ventrolateral reticulospinal tract	Efferent
19	WM left lateral vestibulospinal tract	Efferent
20	WM right lateral vestibulospinal tract	Efferent
21	WM left ventral reticulospinal tract	Efferent
22	WM right ventral reticulospinal tract	Efferent
23	WM left ventral corticospinal tract	Efferent
24	WM right ventral corticospinal tract	Efferent
25	WM left tectospinal tract	Efferent
26	WM right tectospinal tract	Efferent
27	WM left medial reticulospinal tract	Efferent
28	WM right medial reticulospinal tract	Efferent
29	WM left medial longitudinal fasciculus	Both
30	WM right medial longitudinal fasciculus	Both

### Statistical analysis

**FIG. 3.**
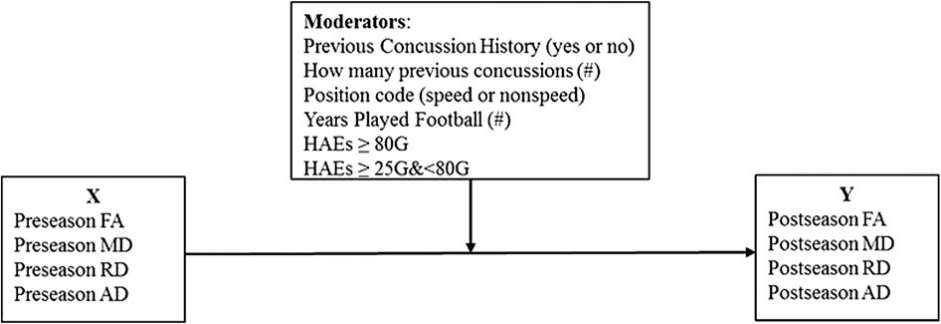
Moderation model for white matter ROIs pre-season to post-season. AD, axial diffusivity; FA, fractional anisotropy; HAE, head acceleration event; MD, mean diffusivity; RD, radial diffusivity; ROI, region of interest.

All statistical analyses were performed using SPSS V25 (IBM Corp., Somers, NY, USA). Average FA, AD, MD, and RD values were calculated for each of the 30 segmented regions of interest (ROIs) for each participant at both pre-season and post-season time-points. Paired *t* tests were run to evaluate within-person changes from pre-season to post-season for each ROI. Additional paired *t* tests were run on all white matter ROIs (1–30) collectively, as well as on efferent and afferent pathway tracts. Independent *t* tests were run to examine differences in two primary grouping variables: previous concussion history (yes or no) and position category (speed or non-speed). These were also run from pre-season to post-season using individual ROIs and the grouping of white matter ROIs (see Supplementary Appendix S1, and Supplementary Appendix S1 Tables S1A and S1B therein). Lastly, moderation regression analyses were run by calculating 95% confidence intervals (CIs) and using bootstrapping with 5000 resamples via the Process procedure in SPSS (Fig. 3).^23^ For moderation analyses, the interaction term of X by M was used to determine significance (*p* < 0.05).

## Results

Fifteen participants were included in the study and were 20.4 ± 1.45 years old. All were right-handed, and all played football for an average of 11.25 ± 3.92 years. Of these participants, 9 players had no history of previous concussion, whereas 6 had a previously medically diagnosed concussion (1 concussion, *n* = 4; 2 concussions, *n* = 2). By position, 9 players were considered non-speed (offensive and defensive lineman) and 6 players were considered speed (wide receivers, running backs, linebackers, cornerbacks, etc.). 

HAE assessment data were collected over the course of the season (Table 2). Impact events were collected during practices and assessed using thresholds of ≥25 g and <80 g, and ≥80 g to quantify impacts likely relevant to brain health.^24^

**Table 2.** Accelerometer Data for All Participants (*n* = 15)

Participant	Position	Monitored sessions (53 total)	Total ≥80 g	Total ≥25 g and <80 g
1^a^	Speed	27	0	17
2	Non-speed	42	3	95
3	Non-speed	37	5	116
4	Non-speed	38	7	180
5	Speed	45	5	101
6	Non-speed	30	5	221
7	Speed	43	13	110
8	Speed	51	2	124
9	Non-speed	37	4	155
10^b^	Non-speed	2	2	9
11	Non-speed	36	2	99
12	Speed	45	1	151
13	Non-speed	48	7	301
14	Speed	37	13	205
15	Non-speed	39	2	315

^a^ Players who missed part of the season due to a non-neurological injury.

^b^ Players whose season ended early due to a non-neurological injury

Overall individual ROI analysis showed no significant change in FA, AD, MD, and RD from pre-season to post-season, with a significant decrease in AD only of the right spino-olivary tract (*p* = 0.026; see Supplementary Appendix S1 Tables S2A–D for each individual ROI paired *t* test). However, characterization of white matter tracts (ROIs 1–30) as a whole revealed significant changes from pre-season to post-season (Table 3) in the form of decreased FA (*p* = 0.005) with increased MD (*p* = 0.05) and RD (*p* = 0.004). Moreover, significant decreases in FA (Table 3) were seen in both the composite subdivision of afferent neurons (*p* = 0.012) and efferent neurons (*p* = 0.041) with significant increase seen in RD for efferent neurons only (*p* = 0.034).

**Table 3.** Paired *t* Test Pre-Season to Post-Season for All White Matter Tracks and All Afferent and Efferent Tracts

		Mean	SD	CI	t	P-value
White matter	Pre FA	0.72961	0.07124	(0.00340, 0.01846)	2.851	0.005^*^
	Post FA	0.71868	0.08393			
	Pre MD	0.00099	0.00020	(-0.00003, -0.00000003)	-1.969	0.050^*^
	Post MD	0.00100	0.00023			
	Pre RD	0.00043	0.00023	(-0.00004, -0.00001)	-2.869	0.004^*^
	Post RD	0.00046	0.00025			
	Pre AD	0.00209	0.00024	(-0.00002, 0.00003)	0.373	0.709
	Post AD	0.00209	0.00028			
Afferent	Pre FA	0.74807	0.04725	(0.00298, 0.02373)	2.545	0.012^*^
	Post FA	0.73471	0.06625			
	Pre MD	0.00095	0.00017	(-0.00004, 0.00001)	-1.048	0.296
	Post MD	0.00096	0.00021			
	Pre RD	0.00036	0.00020	(-0.00005, 0.000004)	-1.699	0.091
	Post RD	0.00039	0.00024			
	Pre AD	0.00212	0.00016	(-0.00002, 0.00004)	0.405	0.686
	Post AD	0.00211	0.00022			
Efferent	Pre FA	0.72257	0.07890	(0.00045, 0.02194)	2.052	0.041^*^
	Post FA	0.71138	0.09212			
	Pre MD	0.00100	0.00023	(-0.00003, 0.000008)	-1.219	0.224
	Post MD	0.00102	0.00025			
	Pre RD	0.00046	0.00024	(-0.00005, -0.000002)	-2.133	0.034^*^
	Post RD	0.00049	0.00026			
	Pre AD	0.00209	0.00027	(-0.00002, 0.00004)	0.809	0.419
	Post AD	0.00208	0.00031			

^*^ Significant (*p* < 0.05).

AD, axial diffusivity; CI, confidence interval; FA, fractional anisotropy; MD, mean diffusivity; Post, post-season; Pre, pre-season; RD, radial diffusivity; SD, standard deviation.

Previous concussion history (both “yes” versus “no” and number of previous concussions), years playing football, position code, and number of HAEs ≥80 g, and ≥25g and <80g, were examined as independent moderators of the relation between pre-season to post-season imaging white matter values.

**FIG. 4.**
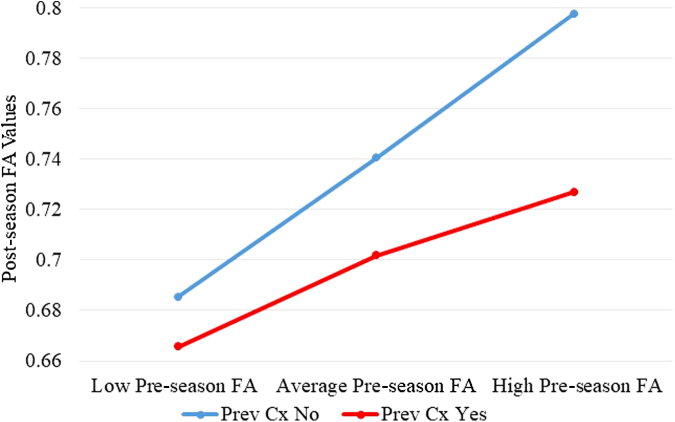
Moderation results of pre-season and post-season FA values with previous concussion history. FA, fractional anisotropy, Prev Cx, previous concussion.

Pre-season white matter FA and previous concussion were entered into the regression analysis and the interaction term explained a significant increase in the variance of post-season FA values (R^2^ = 0.2683, F[3, 446] = 54.5045, *p* < 0.001). Thus, previous concussion history (*b* = 0.1286, *t*[446] = 1.7239, *p* = 0.08) was a significant moderator (*b* = −0.2265, *t*[446] = −2.2393, *p* = 0.0256) of the relationship between pre-season FA (*b* = 0.2559, *t*[446] = 5.9197, *p* < 0.001) and post-season FA values. For both players with (*b* = 0.4293, *t* = 5.2528, *p* < 0.001) and without history of concussion (*b* = 0.6558, *t* = 11.0095, *p* < 0.001), there was a positive relationship between history and post-season FA levels (Fig. 4).

**FIG. 5.**
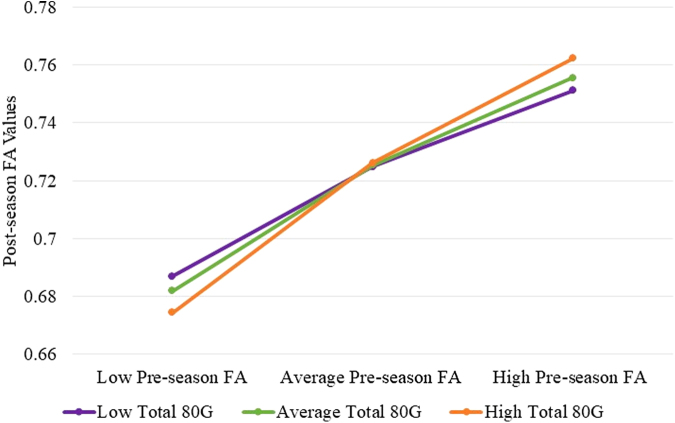
Moderation results of pre-season and post-season FA values with total HAEs ≥80 G. FA, fractional anisotropy; HAE, head acceleration event.

Pre-season white matter FA and HAEs ≥80 g throughout the season were entered into the regression analysis and the interaction term explained a significant increase in the variance of post-season FA values (R^2^ = 0.2230, F[3, 446] = 42.6737, *p* < 0.001). Thus, HAEs ≥80 g (*b* = −0.0241, *t*[446] = −2.4661, *p* = 0.014) was a significant moderator (*b* = 0.0330, *t*[446] = 2.4287, *p* = 0.0155) of the relationship between pre-season FA (*b* = 0.3833, *t*[446] = 7.3674, *p* < 0.001) and post-season FA values. For both players with low (*b* = 0.4493, *t* = 7.2470, *p* < 0.001), average (*b* = 0.5154, *t* = 10.0839, *p* < 0.001), and high numbers of 80 g HAEs (*b* = 0.6144, *t* = 10.3325, *p* < 0.001), there was a positive relationship between impacts and post-season FA levels (Fig. 5).

Although not reaching statistical significance (*p* > 0.05), FA, AD, RD, and MD showed a moderate relationship for number of prior concussions (FA: *p* = 0.065; AD: *p* = 0.052; RD: *p* = 0.051; MD: *p* = 0.063) and FA and AD showed a moderate relationship with HAEs >25 g (FA: *p* = 0.098; AD: *p* = 0.047).

## Discussion

In this study, we examined the effects of exposure to repetitive HAEs on spinal cord integrity over the course of a single season of NCAA football. Specifically, athletes underwent pre-season and post-season DTI scans to evaluate white matter integrity of the cervical spinal cord. There are several findings of interest. First, we documented a significant reduction in AD in the right spino-olivary tract sustained during the course of a competitive football season. Second, looking at the global white matter integrity of the cervical spinal cord, we observed a substantial decrease in FA and increase in MD and RD, which was not specifically confined to either afferent or efferent pathways. Third, these changes from pre-season to post-season were moderated by previous concussion history and exposure to impacts. It is important to note that no participants under study received a clinically diagnosed SRC during the course of the study. Yet, despite not suffering SRC, we observed measurable changes in the white matter integrity of the cervical spinal cord, implicating injury from repetitive HAEs may extend outside the brain to include the entirety of the CNS.

Previous research using DTI in SRC has revealed widespread white matter abnormalities in the brain, as reported by changes in FA, MD, AD, and RD. However, these studies have yielded mixed findings highlighting potential confounds including timing of DTI acquisition.^12^ Consistent with our observed reduction in FA and increased diffusivity measures, studies employing a comparable experimental setup, with DTI being performed at pre-season and post-season intervals, have also reported decreases in FA^25,26^ along with increases in MD and RD indices.^25,27,28^ Nonetheless, all of these studies have focused exclusively on the brain, omitting evaluation of spinal cord white matter. DTI has also been used to assess white matter changes in spinal cord injuries (SCIs) and has demonstrated significant decreases in FA and increased RD.^29–31^ In addition, DTI of chronic SCI has revealed significant reductions in FA and increased diffusivity indices.^31,32^ Following neck flexion and extension there is potential for injury to occur from disc herniation and ligamentous injury that may influence clinical symptoms, recovery, and molecular diffusion.^31^ However, anatomical MRIs of these participants revealed no radiological findings for disc herniation or suggested concomitant soft-tissue injury.

Demyelination, remyelination, axonal loss, and atrophy are all associated with changes in molecular diffusion in chronic SCI, which may also be a result of exposure to HAEs over the course of a season or career.^33–35^ DTI analysis revealed a widespread distribution of affected spinal cord tracts including afferent and efferent pathways, which suggests that the hallmark diffuse axonal injury (DAI) seen in concussive injuries^36^ may not be localized to just the brain. Given the parallel between the mechanism of injury in both SRC and WAD, injury to the spinal cord is expected; however, we cannot rule out transneuronal degeneration of the spinal cord as an evolution of injury from repetitive HAEs.^37^ Moreover, injury to the spinal cord from HAEs may reflect a similar relationship between subconcussive brain injury and SRC, but under the threshold to produce a spinal cord concussion.^38^

The potential for HAEs to infer injury to the spinal cord has major implications on the diagnosis, management, and monitoring of symptoms resolution in SRC. Missed diagnoses of concussive injuries in WAD and SCI is a concern and may occur in upwards of 74% of cases.^39^ Consequently, it is likely that injury to the spinal cord is overlooked in SRC. Here we observed a significant change in the right spino-olivary tract, which can have anterograde and retrograde effects on the afferent transmission of important balance and proprioception information from tendons and muscles to the cerebellum.^40^

Additionally, trending effects were seen in the right ventral corticospinal (decrease in FA, increase in RD) and right medial reticulospinal (increase in RD) tracts. The ventral corticospinal tract, an efferent pathway, is crucial for control of axial and proximal limb muscles involved in posture and balance, whereas the medial reticulospinal tract, an efferent pathway, is crucial for regulation of extensor, or proximal, muscle function to maintain posture and balance.^41^ In combination, the involvement of these three pathways suggests a potential role of balance and postural stability being one of the processes most negatively affected after exposure to HAEs. Given the recommended evaluation of neurocognitive functions, such as balance and reaction time,^1^ spinal cord involvement may have a direct effect on outcomes, performance, and success rate on these tests^42^ and warrants further exploration. Moreover, injury to the spinal cord may shed light into post-concussive syndrome (PCS) and long-term disabilities^43^ associated with SRC and symptoms resolution.

Interestingly, moderation analyses revealed that years playing football and position code, both known to increase exposure to HAEs and risk of long-term deficits,^44–46^ did not moderate the changes on any DTI metric. However, concussion history and exposure to impacts ≥80 g did significantly moderate these changes over the season, but only for FA values. These findings suggest that using exposure to high-intensity impacts and previous concussion history, as well as FA values, could be potentially useful clinical factors to consider both in terms of an individual's risk and outcome after exposure. Further, given that those individuals with a previous history of concussion had worse imaging outcomes at post-season, this could suggest an accumulative effect of damage that continues to be compounded with exposure to HAEs after diagnosis.

The etiology of PCS remains unclear with most researchers adopting the view that it is a compilation of many factors that span the spectrum from biological to psychological.^47^ PCS is one of the most controversial syndromes in sports medicine today, with a constellation of symptoms^48^ that may also mimic traumatic cervical injuries resulting in chronic pain, headache, and neck pain.^49^ This presents many challenges for the clinician and highlights the important need for a more systematic approach in the evaluation of PCS to better handle the possible contributing differential diagnoses, co-morbidities, and psychological factors.^49^ The neural mechanisms, pathophysiology, and other contributing factors to the symptomology of concussion are poorly understood and could be caused by abnormalities anywhere in the CNS.^50^

It should be noted that this study has limitations. It involved a small sample size of only male, football athletes from one collegiate team. This limits generalizability and future work should be done to replicate these findings, as well as to expand on this work in different ages and sexes. Additionally, it was the study was conducted over a short time frame, so more studies involving longitudinal data are warranted to better identify if the compromised cervical spinal cord seen here worsens over time with consistent exposure or the extent of recovery in the absence of HAEs.

## Conclusion

In a study of collegiate football players, it was found that there are significant changes in white matter integrity of the spinal cord with exposure to repetitive HAEs. Despite no diagnosed SRC, exposure to these repetitive impacts suggests damage to the cervical spinal cord; however, the extent and permanence of this damage is still unknown. Future work should be taken to replicate these findings and relate them to functional outcomes, especially the potential link to postural instability. These findings suggest that injury from repetitive HAEs or SRC may extend outside of the brain to include the entire CNS and clinical consideration of these other areas, particularly the cervical spinal cord, should be considered.

## Supplementary Material

Supplemental data

Supplemental data

## Supplementary Material

Supplementary Appendix S1

Supplementary Appendix S1 Table S1A

Supplementary Appendix S1 Table S1B

Supplementary Appendix S1 Table S2A

Supplementary Appendix S1 Table S2B

Supplementary Appendix S1 Table S2C

Supplementary Appendix S1 Table S2D

## Abbreviations Used

2D: two-dimensional
3D: three-dimensional
AD: axial diffusivity
CI: confidence interval
CNS: central nervous system
DAI: diffuse axonal injury
DTI: diffusion tensor imaging
FA: fractional anisotropy
HAE: head acceleration event
MD: mean diffusivity
MRI: magnetic resonance imaging
NCAA: National Collegiate Athletic Association
PCS: post-concussive syndrome
RD: radial diffusivity
ROI: regions of interest
SCI: spinal cord injury
SD: standard deviation
SPAIR: spectral attenuated inversion recovery
SRC: sports-related concussion
TA: acquisition time
TE: echo time
TR: repetition time
WAD: whiplash-associated disorder
