# Supplementary material for: Changes in White Matter of the Cervical Spinal Cord after a Single Season of Collegiate Football
Source: Neurotrauma Rep. 2021 Feb 17;2(1):84–93. doi: 10.1089/neur.2020.0035 (PMC8240824; doi:10.1089/neur.2020.0035)
Supplement: Supplemental data [file Supp_Data.docx]

**Supplemental File 1:**

Exploratory independent t-tests were run to examine differences in two primary grouping variables: previous concussion history (yes or no) and position category (speed or nonspeed). For each individual ROI, preseason to postseason changes were evaluated and significant differences existed for those with and without a previous concussion history. Those players with no previous concussion history only had significant changes from preseason to postseason in FA13 (left spinal lemniscus; up; p=0.032), MD18 (right ventrolateral reticulospinal; down; p=0.037), RD23 (left ventral corticospinal; up; p=0.018), and AD16 (right spino-olivary; down; p=0.041). Those players with a history of previous concussive injury only had significant changes from preseason to postseason in RD5 (left lateral corticospinal; up; p=0.033) and RD24 (right ventral corticospinal; up; p=0.002). Globally, in all white matter tracts, there were no significant changes pre to post season for those individuals with no history of concussion (p>0.05). In those players with a history of concussion, there were global significant decreases in FA (p<0.001), increases in MD (p<0.001), and increases in RD (p<0.001).

Additionally, for each individual ROI, preseason to postseason changes were evaluated and significant differences existed for those playing nonspeed versus speed positions. Nonspeed players only had significant changes from preseason to postseason in AD12 (right lateral reticulospinal; up; p=0.010). Speed players however, had significant changes from preseason to postseason in FA16 (right spino-olivary; down; p=0.011), AD7 (left ventral spinocerebellar; down; p=0.002), and AD13 (left spinal lemniscus; down; p=0.002). Globally, in all white matter tracts, there were significant changes pre to post season for those speed players having a significant decrease in AD (p<0.001) and for nonspeed players showing significant global decreases in FA (p=0.010), increases in MD (p=0.002), increases in RD (p=0.007), and decreases in AD (p=0.011).

Differences between groups at each time point for all white matter ROIs are presented for previous concussion history (eTable1A) and position (eTable1B).

| eTable1A. Group Statistics for White Matter based on Previous History of Concussion (Yes or No) | | | | | | | | |
| --- | --- | --- | --- | --- | --- | --- | --- | --- |
|  | | | **N** | **Mean** | **Std. Dev** | **t** | **Sig. (2-tailed)** |  |
| White Matter | Pre FA | no | 270 | 0.7219 | 0.0737 | -2.836 | 0.005* |  |
|  |  | yes | 180 | 0.7412 | 0.0659 |  |  |  |
|  | Pre MD | no | 270 | 0.0010 | 0.0002 | 1.146 | 0.252 |  |
|  |  | yes | 180 | 0.0010 | 0.0002 |  |  |  |
|  | Pre RD | no | 270 | 0.0004 | 0.0002 | 1.69 | 0.092 |  |
|  |  | yes | 180 | 0.0004 | 0.0002 |  |  |  |
|  | Pre AD | no | 270 | 0.0021 | 0.0002 | -0.257 | 0.797 |  |
|  |  | yes | 180 | 0.0021 | 0.0002 |  |  |  |
|  | Post FA | no | 270 | 0.7293 | 0.0880 | 3.329 | 0.001* |  |
|  |  | yes | 180 | 0.7027 | 0.0748 |  |  |  |
|  | Post MD | no | 270 | 0.0010 | 0.0002 | -1.685 | 0.093 |  |
|  |  | yes | 180 | 0.0010 | 0.0003 |  |  |  |
|  | Post RD | no | 270 | 0.0004 | 0.0002 | -2.207 | 0.028* |  |
|  |  | yes | 180 | 0.0005 | 0.0003 |  |  |  |
|  | Post AD | no | 270 | 0.0021 | 0.0003 | -0.237 | 0.813 |  |
|  |  | yes | 180 | 0.0021 | 0.0003 |  |  |  |
| **significant (p<0.05)* | | | | | | | | |

| eTable1B. Group Statistics for White Matter based on Position Code (Nonspeed vs Speed) | | | | | | | | |
| --- | --- | --- | --- | --- | --- | --- | --- | --- |
|  | | | N | Mean | Std. Deviation | t | Sig. (2-tailed) |  |
| White Matter | Pre FA | nonspeed | 270 | 0.7259 | 0.0692 | -1.364 | 0.173 |  |
|  |  | speed | 180 | 0.7352 | 0.0740 |  |  |  |
|  | Pre MD | nonspeed | 270 | 0.0010 | 0.0002 | 0.435 | 0.664 |  |
|  |  | speed | 180 | 0.0010 | 0.0002 |  |  |  |
|  | Pre RD | nonspeed | 270 | 0.0004 | 0.0002 | 0.956 | 0.340 |  |
|  |  | speed | 180 | 0.0004 | 0.0002 |  |  |  |
|  | Pre AD | nonspeed | 270 | 0.0021 | 0.0002 | -0.702 | 0.483 |  |
|  |  | speed | 180 | 0.0021 | 0.0002 |  |  |  |
|  | Post FA | nonspeed | 270 | 0.7133 | 0.0807 | -1.659 | 0.098 |  |
|  |  | speed | 180 | 0.7267 | 0.0881 |  |  |  |
|  | Post MD | nonspeed | 270 | 0.0010 | 0.0002 | 2.319 | 0.021* |  |
|  |  | speed | 180 | 0.0010 | 0.0002 |  |  |  |
|  | Post RD | nonspeed | 270 | 0.0005 | 0.0003 | 1.481 | 0.139 |  |
|  |  | speed | 180 | 0.0004 | 0.0003 |  |  |  |
|  | Post AD | nonspeed | 270 | 0.0021 | 0.0003 | 3.186 | 0.002* |  |
|  |  | speed | 180 | 0.0020 | 0.0003 |  |  |  |
| **significant (p<0.05)* | | | | | | | | |
