# Supplementary material for: Changes in White Matter of the Cervical Spinal Cord after a Single Season of Collegiate Football
Source: Neurotrauma Rep. 2021 Feb 17;2(1):84–93. doi: 10.1089/neur.2020.0035 (PMC8240824; doi:10.1089/neur.2020.0035)
Supplement: Supplemental data [file Supp_TableS2A1-S1A4.docx]

| eTable 2A. Fractional Anisotropy (FA) values at preseason (FA.#) and postseason (PostFA.#) and paired t-test results for each individual ROI. | | | | | | | | | | |
| --- | --- | --- | --- | --- | --- | --- | --- | --- | --- | --- |
|  | **Mean** | **Std. Deviation** | **t** | **Sig. (2-tailed)** |  | **Mean** | **Std. Deviation** | **t** | **Sig. (2-tailed)** |  |
| FA.1 | 0.7731 | 0.0436 | 1.411 | 0.180 | **FA.16** | 0.7329 | 0.0402 | 1.456 | 0.167 |  |
| PostFA.1 | 0.7044 | 0.2017 |  |  | **PostFA.16** | 0.6596 | 0.2014 |  |  |  |
| FA.2 | 0.7670 | 0.0455 | 1.355 | 0.197 | **FA.17** | 0.7637 | 0.0617 | 1.008 | 0.331 |  |
| PostFA.2 | 0.7023 | 0.2024 |  |  | **PostFA.17** | 0.7105 | 0.2056 |  |  |  |
| FA.3 | 0.7671 | 0.0379 | 1.284 | 0.220 | **FA.18** | 0.7622 | 0.0462 | 0.985 | 0.341 |  |
| PostFA.3 | 0.7052 | 0.1988 |  |  | **PostFA.18** | 0.7093 | 0.2035 |  |  |  |
| FA.4 | 0.7662 | 0.0367 | 1.348 | 0.199 | **FA.19** | 0.6956 | 0.0859 | 0.968 | 0.349 |  |
| PostFA.4 | 0.7022 | 0.1990 |  |  | **PostFA.19** | 0.6350 | 0.1889 |  |  |  |
| FA.5 | 0.7312 | 0.0359 | 1.191 | 0.254 | **FA.20** | 0.7033 | 0.0653 | 1.164 | 0.264 |  |
| PostFA.5 | 0.6723 | 0.1944 |  |  | **PostFA.20** | 0.6473 | 0.1870 |  |  |  |
| FA.6 | 0.7213 | 0.0438 | 1.243 | 0.234 | **FA.21** | 0.6877 | 0.0927 | 1.328 | 0.205 |  |
| PostFA.6 | 0.6666 | 0.1914 |  |  | **PostFA.21** | 0.6094 | 0.2176 |  |  |  |
| FA.7 | 0.7274 | 0.0649 | 1.019 | 0.326 | **FA.22** | 0.7394 | 0.0801 | 1.290 | 0.218 |  |
| PostFA.7 | 0.6698 | 0.2003 |  |  | **PostFA.22** | 0.6561 | 0.2192 |  |  |  |
| FA.8 | 0.7558 | 0.0477 | 1.350 | 0.198 | **FA.23** | 0.6205 | 0.0388 | 0.934 | 0.366 |  |
| PostFA.8 | 0.6819 | 0.2015 |  |  | **PostFA.23** | 0.5816 | 0.1783 |  |  |  |
| FA.9 | 0.7832 | 0.0607 | 1.232 | 0.238 | **FA.24** | 0.6467 | 0.0845 | 1.945 | 0.072 |  |
| PostFA.9 | 0.7217 | 0.2055 |  |  | **PostFA.24** | 0.5603 | 0.1670 |  |  |  |
| FA.10 | 0.7878 | 0.0543 | 1.473 | 0.163 | **FA.25** | 0.6695 | 0.0622 | 0.811 | 0.431 |  |
| PostFA.10 | 0.7149 | 0.2058 |  |  | **PostFA.25** | 0.6301 | 0.1939 |  |  |  |
| FA.11 | 0.7511 | 0.0402 | 0.929 | 0.369 | **FA.26** | 0.7258 | 0.0572 | 0.602 | 0.557 |  |
| PostFA.11 | 0.7048 | 0.2044 |  |  | **PostFA.26** | 0.6921 | 0.2027 |  |  |  |
| FA.12 | 0.7748 | 0.0525 | 0.981 | 0.343 | **FA.27** | 0.7112 | 0.0820 | 1.092 | 0.293 |  |
| PostFA.12 | 0.7279 | 0.2089 |  |  | **PostFA.27** | 0.6482 | 0.2069 |  |  |  |
| FA.13 | 0.7548 | 0.0227 | 1.120 | 0.281 | **FA.28** | 0.7393 | 0.0985 | 1.580 | 0.137 |  |
| PostFA.13 | 0.6991 | 0.1966 |  |  | **PostFA.28** | 0.6583 | 0.2025 |  |  |  |
| FA.14 | 0.7604 | 0.0338 | 1.350 | 0.199 | **FA.29** | 0.6826 | 0.0793 | 0.561 | 0.583 |  |
| PostFA.14 | 0.6979 | 0.1976 |  |  | **PostFA.29** | 0.6557 | 0.1982 |  |  |  |
| FA.15 | 0.6931 | 0.0460 | 1.218 | 0.243 | **FA.30** | 0.7075 | 0.0764 | 1.101 | 0.289 |  |
| PostFA.15 | 0.6316 | 0.1879 |  |  | **PostFA.30** | 0.6593 | 0.1951 |  |  |  |

| eTable 2B. Mean Diffusivity (MD) values at preseason (MD.#) and postseason (PostMD.#) and paired t-test results for each individual ROI. | | | | | | | | | | |
| --- | --- | --- | --- | --- | --- | --- | --- | --- | --- | --- |
|  | **Mean** | **Std. Deviation** | **t** | **Sig. (2-tailed)** |  | **Mean** | **Std. Deviation** | **t** | **Sig. (2-tailed)** |  |
| MD.1 | 0.0010 | 0.0001 | -0.875 | 0.396 | **MD.16** | 0.0009 | 0.0001 | 0.649 | 0.527 |  |
| PostMD.1 | 0.0010 | 0.0001 |  |  | **PostMD.16** | 0.0009 | 0.0002 |  |  |  |
| MD.2 | 0.0010 | 0.0001 | -1.259 | 0.229 | **MD.17** | 0.0009 | 0.0001 | 1.427 | 0.176 |  |
| PostMD.2 | 0.0011 | 0.0001 |  |  | **PostMD.17** | 0.0009 | 0.0001 |  |  |  |
| MD.3 | 0.0010 | 0.0001 | -0.959 | 0.354 | **MD.18** | 0.0009 | 0.0001 | -0.337 | 0.741 |  |
| PostMD.3 | 0.0011 | 0.0001 |  |  | **PostMD.18** | 0.0010 | 0.0001 |  |  |  |
| MD.4 | 0.0010 | 0.0001 | -0.905 | 0.381 | **MD.19** | 0.0013 | 0.0001 | 0.335 | 0.742 |  |
| PostMD.4 | 0.0010 | 0.0001 |  |  | **PostMD.19** | 0.0013 | 0.0002 |  |  |  |
| MD.5 | 0.0011 | 0.0001 | -1.203 | 0.249 | **MD.20** | 0.0012 | 0.0001 | 0.077 | 0.939 |  |
| PostMD.5 | 0.0011 | 0.0001 |  |  | **PostMD.20** | 0.0012 | 0.0002 |  |  |  |
| MD.6 | 0.0011 | 0.0000 | -1.481 | 0.161 | **MD.21** | 0.0006 | 0.0003 | -0.759 | 0.461 |  |
| PostMD.6 | 0.0011 | 0.0001 |  |  | **PostMD.21** | 0.0007 | 0.0004 |  |  |  |
| MD.7 | 0.0007 | 0.0001 | -0.183 | 0.857 | **MD.22** | 0.0005 | 0.0003 | -0.006 | 0.995 |  |
| PostMD.7 | 0.0007 | 0.0002 |  |  | **PostMD.22** | 0.0005 | 0.0003 |  |  |  |
| MD.8 | 0.0006 | 0.0002 | -0.450 | 0.660 | **MD.23** | 0.0013 | 0.0001 | -0.508 | 0.620 |  |
| PostMD.8 | 0.0007 | 0.0002 |  |  | **PostMD.23** | 0.0013 | 0.0002 |  |  |  |
| MD.9 | 0.0010 | 0.0001 | -0.407 | 0.690 | **MD.24** | 0.0011 | 0.0002 | -1.257 | 0.229 |  |
| PostMD.9 | 0.0010 | 0.0001 |  |  | **PostMD.24** | 0.0012 | 0.0002 |  |  |  |
| MD.10 | 0.0010 | 0.0001 | -1.410 | 0.180 | **MD.25** | 0.0012 | 0.0001 | -0.405 | 0.692 |  |
| PostMD.10 | 0.0011 | 0.0001 |  |  | **PostMD.25** | 0.0012 | 0.0001 |  |  |  |
| MD.11 | 0.0009 | 0.0001 | -0.364 | 0.721 | **MD.26** | 0.0011 | 0.0001 | 1.103 | 0.289 |  |
| PostMD.11 | 0.0009 | 0.0001 |  |  | **PostMD.26** | 0.0010 | 0.0001 |  |  |  |
| MD.12 | 0.0009 | 0.0001 | -0.043 | 0.966 | **MD.27** | 0.0010 | 0.0001 | -0.293 | 0.774 |  |
| PostMD.12 | 0.0009 | 0.0001 |  |  | **PostMD.27** | 0.0010 | 0.0001 |  |  |  |
| MD.13 | 0.0010 | 0.0001 | -0.494 | 0.629 | **MD.28** | 0.0010 | 0.0001 | -1.470 | 0.164 |  |
| PostMD.13 | 0.0011 | 0.0001 |  |  | **PostMD.28** | 0.0010 | 0.0001 |  |  |  |
| MD.14 | 0.0010 | 0.0001 | -1.422 | 0.177 | **MD.29** | 0.0010 | 0.0001 | -1.151 | 0.269 |  |
| PostMD.14 | 0.0011 | 0.0001 |  |  | **PostMD.29** | 0.0010 | 0.0001 |  |  |  |
| MD.15 | 0.0010 | 0.0001 | 0.511 | 0.617 | **MD.30** | 0.0010 | 0.0001 | -1.443 | 0.171 |  |
| PostMD.15 | 0.0010 | 0.0002 |  |  | **PostMD.30** | 0.0011 | 0.0001 |  |  |  |

| eTable 2C. Radial Diffusivity (RD) values at preseason (RD.#) and postseason (PostRD.#) and paired t-test results for each individual ROI. | | | | | | | | | | |
| --- | --- | --- | --- | --- | --- | --- | --- | --- | --- | --- |
|  | **Mean** | **Std. Deviation** | **t** | **Sig. (2-tailed)** |  | **Mean** | **Std. Deviation** | **t** | **Sig. (2-tailed)** |  |
| RD.1 | 0.0004 | 0.0001 | -1.368 | 0.193 | **RD.16** | 0.0004 | 0.0001 | -0.188 | 0.853 |  |
| PostRD.1 | 0.0005 | 0.0001 |  |  | **PostRD.16** | 0.0004 | 0.0002 |  |  |  |
| RD.2 | 0.0004 | 0.0001 | -1.558 | 0.142 | **RD.17** | 0.0004 | 0.0001 | 0.509 | 0.619 |  |
| PostRD.2 | 0.0005 | 0.0001 |  |  | **PostRD.17** | 0.0004 | 0.0001 |  |  |  |
| RD.3 | 0.0004 | 0.0001 | -0.968 | 0.350 | **RD.18** | 0.0004 | 0.0001 | -0.466 | 0.648 |  |
| PostRD.3 | 0.0005 | 0.0001 |  |  | **PostRD.18** | 0.0005 | 0.0001 |  |  |  |
| RD.4 | 0.0004 | 0.0001 | -1.063 | 0.306 | **RD.19** | 0.0007 | 0.0002 | -0.397 | 0.697 |  |
| PostRD.4 | 0.0005 | 0.0001 |  |  | **PostRD.19** | 0.0007 | 0.0002 |  |  |  |
| RD.5 | 0.0005 | 0.0001 | -1.344 | 0.200 | **RD.20** | 0.0007 | 0.0001 | -0.618 | 0.547 |  |
| PostRD.5 | 0.0006 | 0.0001 |  |  | **PostRD.20** | 0.0007 | 0.0002 |  |  |  |
| RD.6 | 0.0005 | 0.0001 | -1.190 | 0.254 | **RD.21** | 0.0001 | 0.0003 | -0.996 | 0.336 |  |
| PostRD.6 | 0.0006 | 0.0001 |  |  | **PostRD.21** | 0.0002 | 0.0005 |  |  |  |
| RD.7 | 0.0001 | 0.0002 | -0.384 | 0.706 | **RD.22** | -0.0001 | 0.0003 | -0.474 | 0.643 |  |
| PostRD.7 | 0.0001 | 0.0003 |  |  | **PostRD.22** | 0.0000 | 0.0003 |  |  |  |
| RD.8 | 0.0000 | 0.0002 | -0.825 | 0.423 | **RD.23** | 0.0007 | 0.0001 | -0.671 | 0.513 |  |
| PostRD.8 | 0.0000 | 0.0002 |  |  | **PostRD.23** | 0.0008 | 0.0002 |  |  |  |
| RD.9 | 0.0004 | 0.0001 | -0.894 | 0.386 | **RD.24** | 0.0006 | 0.0002 | -2.065 | 0.058 |  |
| PostRD.9 | 0.0004 | 0.0001 |  |  | **PostRD.24** | 0.0007 | 0.0002 |  |  |  |
| RD.10 | 0.0004 | 0.0001 | -1.638 | 0.124 | **RD.25** | 0.0007 | 0.0001 | 0.020 | 0.984 |  |
| PostRD.10 | 0.0005 | 0.0001 |  |  | **PostRD.25** | 0.0007 | 0.0002 |  |  |  |
| RD.11 | 0.0004 | 0.0001 | -0.088 | 0.931 | **RD.26** | 0.0005 | 0.0001 | 0.657 | 0.522 |  |
| PostRD.11 | 0.0004 | 0.0001 |  |  | **PostRD.26** | 0.0005 | 0.0002 |  |  |  |
| RD.12 | 0.0004 | 0.0000 | 0.190 | 0.852 | **RD.27** | 0.0005 | 0.0001 | -0.683 | 0.506 |  |
| PostRD.12 | 0.0004 | 0.0001 |  |  | **PostRD.27** | 0.0005 | 0.0002 |  |  |  |
| RD.13 | 0.0005 | 0.0000 | -0.779 | 0.449 | **RD.28** | 0.0005 | 0.0001 | -1.926 | 0.075 |  |
| PostRD.13 | 0.0005 | 0.0001 |  |  | **PostRD.28** | 0.0005 | 0.0001 |  |  |  |
| RD.14 | 0.0005 | 0.0001 | -1.656 | 0.120 | **RD.29** | 0.0005 | 0.0001 | -0.283 | 0.781 |  |
| PostRD.14 | 0.0005 | 0.0001 |  |  | **PostRD.29** | 0.0005 | 0.0001 |  |  |  |
| RD.15 | 0.0005 | 0.0001 | 0.123 | 0.904 | **RD.30** | 0.0005 | 0.0001 | -1.144 | 0.272 |  |
| PostRD.15 | 0.0005 | 0.0002 |  |  | **PostRD.30** | 0.0005 | 0.0001 |  |  |  |

| eTable 2D. Axial Diffusivity (AD) values at preseason (AD.#) and postseason (PostAD.#) and paired t-test results for each individual ROI. | | | | | | | | | | |
| --- | --- | --- | --- | --- | --- | --- | --- | --- | --- | --- |
|  | **Mean** | **Std. Deviation** | **t** | **Sig. (2-tailed)** |  | **Mean** | **Std. Deviation** | **t** | **Sig. (2-tailed)** |  |
| AD.1 | 0.0022 | 0.0001 | -0.031 | 0.976 | **AD.16** | 0.0021 | 0.0002 | 2.496 | **0.026** |  |
| PostAD.1 | 0.0022 | 0.0002 |  |  | **PostAD.16** | 0.0020 | 0.0002 |  |  |  |
| AD.2 | 0.0022 | 0.0001 | -0.537 | 0.600 | **AD.17** | 0.0020 | 0.0002 | 0.749 | 0.466 |  |
| PostAD.2 | 0.0022 | 0.0002 |  |  | **PostAD.17** | 0.0019 | 0.0002 |  |  |  |
| AD.3 | 0.0022 | 0.0001 | -0.725 | 0.481 | **AD.18** | 0.0020 | 0.0002 | -0.101 | 0.921 |  |
| PostAD.3 | 0.0022 | 0.0002 |  |  | **PostAD.18** | 0.0020 | 0.0002 |  |  |  |
| AD.4 | 0.0022 | 0.0001 | -0.462 | 0.651 | **AD.19** | 0.0024 | 0.0001 | 1.236 | 0.237 |  |
| PostAD.4 | 0.0022 | 0.0002 |  |  | **PostAD.19** | 0.0023 | 0.0002 |  |  |  |
| AD.5 | 0.0021 | 0.0001 | -0.730 | 0.477 | **AD.20** | 0.0023 | 0.0002 | 0.821 | 0.425 |  |
| PostAD.5 | 0.0022 | 0.0002 |  |  | **PostAD.20** | 0.0023 | 0.0002 |  |  |  |
| AD.6 | 0.0022 | 0.0001 | -1.506 | 0.154 | **AD.21** | 0.0017 | 0.0003 | 0.210 | 0.836 |  |
| PostAD.6 | 0.0022 | 0.0002 |  |  | **PostAD.21** | 0.0017 | 0.0004 |  |  |  |
| AD.7 | 0.0019 | 0.0001 | 0.232 | 0.820 | **AD.22** | 0.0017 | 0.0003 | 0.641 | 0.532 |  |
| PostAD.7 | 0.0019 | 0.0003 |  |  | **PostAD.22** | 0.0016 | 0.0003 |  |  |  |
| AD.8 | 0.0019 | 0.0002 | 0.293 | 0.774 | **AD.23** | 0.0023 | 0.0002 | -0.111 | 0.913 |  |
| PostAD.8 | 0.0019 | 0.0002 |  |  | **PostAD.23** | 0.0024 | 0.0002 |  |  |  |
| AD.9 | 0.0022 | 0.0001 | 0.384 | 0.707 | **AD.24** | 0.0022 | 0.0002 | 0.719 | 0.484 |  |
| PostAD.9 | 0.0022 | 0.0001 |  |  | **PostAD.24** | 0.0022 | 0.0002 |  |  |  |
| AD.10 | 0.0022 | 0.0001 | -0.658 | 0.521 | **AD.25** | 0.0022 | 0.0002 | -0.742 | 0.471 |  |
| PostAD.10 | 0.0022 | 0.0001 |  |  | **PostAD.25** | 0.0022 | 0.0002 |  |  |  |
| AD.11 | 0.0018 | 0.0001 | -0.596 | 0.560 | **AD.26** | 0.0021 | 0.0002 | 1.072 | 0.302 |  |
| PostAD.11 | 0.0018 | 0.0003 |  |  | **PostAD.26** | 0.0021 | 0.0002 |  |  |  |
| AD.12 | 0.0019 | 0.0002 | -0.233 | 0.819 | **AD.27** | 0.0020 | 0.0002 | 0.479 | 0.639 |  |
| PostAD.12 | 0.0019 | 0.0002 |  |  | **PostAD.27** | 0.0020 | 0.0002 |  |  |  |
| AD.13 | 0.0021 | 0.0001 | -0.058 | 0.955 | **AD.28** | 0.0020 | 0.0002 | -0.396 | 0.698 |  |
| PostAD.13 | 0.0021 | 0.0002 |  |  | **PostAD.28** | 0.0020 | 0.0002 |  |  |  |
| AD.14 | 0.0021 | 0.0001 | -0.928 | 0.369 | **AD.29** | 0.0020 | 0.0002 | -1.531 | 0.148 |  |
| PostAD.14 | 0.0022 | 0.0001 |  |  | **PostAD.29** | 0.0020 | 0.0003 |  |  |  |
| AD.15 | 0.0021 | 0.0001 | 0.864 | 0.402 | **AD.30** | 0.0020 | 0.0001 | -1.623 | 0.127 |  |
| PostAD.15 | 0.0021 | 0.0002 |  |  | **PostAD.30** | 0.0021 | 0.0002 |  |  |  |
